# Interindividual Variability in the Bioavailability of Gabapentin Enacarbil Extended Release in Healthy Adults: An Analysis of Data From 6 Phase I Studies

**DOI:** 10.1097/FTD.0000000000000935

**Published:** 2021-11-01

**Authors:** Ritu Lal, Aaron Ellenbogen, Barry Gidal

**Affiliations:** * GEn1E Lifesciences, Palo Alto, California;; † Quest Research Institute, Farmington Hills, Michigan;; ‡ Michigan Institute for Neurological Disorders, Farmington Hills, Michigan; and; § School of Pharmacy, University of Wisconsin, Madison, Wisconsin.

**Keywords:** absorption, bioavailability, gabapentin, gabapentin enacarbil, pharmacokinetics

## Abstract

The absorption and bioavailability of oral gabapentin are associated with a high degree of interindividual variability. Gabapentin enacarbil, a prodrug of gabapentin, is well absorbed and provides sustained, dose-proportional exposure to gabapentin. The aim of this analysis was to describe the interindividual variability in the bioavailability of gabapentin after gabapentin enacarbil administration in healthy subjects.

**Methods:**

Gabapentin pharmacokinetic (PK) parameters after an oral dose of gabapentin enacarbil 1200 mg (2 600-mg tablets) were compared across 6 phase I studies in healthy adults (n = 12 per study). The distribution of bioavailability values was assessed in all studies.

**Results:**

The mean PK parameters of gabapentin were consistent across the trials: maximum concentration range: 6.4–7.9 μg/mL, time to maximum concentration range: 5.2–8.2 hours, area under the plasma–concentration curve extrapolated from time 0 to infinity or at steady state range: 70.8–109.4 μg·h/mL, and bioavailability range: 64.8%–82.9%. Overall, the mean bioavailability was 74.1% (SD, 14.1; coefficient of variation, 19.1%). Individual bioavailability across all studies ranged from 42% to 100%.

**Conclusions:**

Gabapentin PK after gabapentin enacarbil administration was consistent across studies, with low interindividual variability in bioavailability. Gabapentin enacarbil may provide more consistent and predictable exposure to gabapentin than oral gabapentin formulations.

## BACKGROUND

Gabapentin enacarbil is a prodrug of gabapentin, a synthetic analog of γ-aminobutyric acid used in the treatment of several neuropathological disorders.^1–3^ In the United States, gabapentin enacarbil (Horizant; Arbor Pharmaceuticals, Atlanta, GA) extended release is approved for the treatment of adults with moderate-to-severe restless legs syndrome (RLS) and for the management of postherpetic neuralgia (PHN).^4^ Formulations of gabapentin approved in the United States include gabapentin immediate release (Neurontin; Pfizer, New York, NY)^5^ for the treatment of PHN and as adjunctive therapy for partial-onset seizures and gastroretentive gabapentin (Gralise; Depomed, Menlo Park, CA)^6^ for the treatment of PHN.

Gabapentin is rapidly absorbed by a low-capacity transporter pathway in the upper small intestine.^7–9^ Saturation of this transporter at clinical doses leads to dose-dependent pharmacokinetics (PK) and bioavailability, and high interpatient variability.^9,10^ As a polar compound, gabapentin is not well absorbed by passive diffusion.^11^ Although the gastroretentive formulation permits longer and more gradual release of gabapentin compared with the immediate-release formulation, absorption remains limited.^12^ Thus, plasma exposure to gabapentin after oral dosing is highly variable, making it difficult to determine the concentration resulting from a given dose, potentially resulting in suboptimal drug exposure in some patients.^10^

**FIGURE 1.**
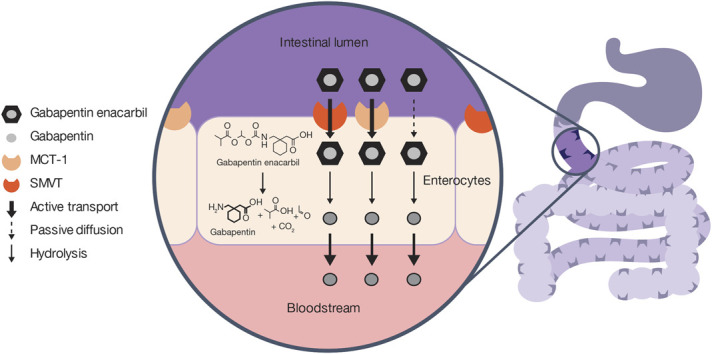
Absorption pathways of gabapentin and gabapentin enacarbil. H, hydrogen; MCT-1, monocarboxylate transporter type 1; N, nitrogen; O, oxygen; OH, hydroxide; SMVT, sodium-dependent multivitamin transporter.

Gabapentin enacarbil is absorbed by high-capacity transporters expressed throughout the intestinal tract, including the monocarboxylate transporter type 1 and the sodium-dependent multivitamin transporter (Fig. 1).^4,13,14^ Some passive absorption of gabapentin enacarbil may also occur due to its lipophilic properties.^14^ After absorption from the intestinal lumen, gabapentin enacarbil undergoes hydrolysis by nonspecific carboxylesterases (primarily in enterocytes and to a lesser extent in the liver) to form gabapentin, the pharmacologically active compound.^4^ While the mechanism of absorption of gabapentin enacarbil differs from that of gabapentin, the distribution and elimination PK of gabapentin subsequently released into the bloodstream are identical; gabapentin, when dosed either as gabapentin or gabapentin enacarbil, is not metabolized and is thus excreted unchanged in the urine.^10,15^ In contrast to gabapentin, the absorption pathway of gabapentin enacarbil does not become saturated at clinically relevant doses, allowing greater bioavailability of the active drug.^13,16^ Gabapentin enacarbil is absorbed through the gastrointestinal (GI) tract and has dose-proportional and predictable PK over a wide dose range, resulting in lower variability of gabapentin exposure compared with the administration of other gabapentin formulations.^16,17^ Hence, the same doses of gabapentin enacarbil and gabapentin formulations cannot be regarded clinically as bioequivalent and interchangeable.

Previous studies have demonstrated a high degree of interindividual variability in the oral bioavailability of gabapentin, even at the same dose^10,17,18^; for example, bioavailability after a single oral 600-mg dose of gabapentin ranged from 5% to 74% in one study. To date, interindividual variability in the bioavailability of gabapentin enacarbil has not been thoroughly and specifically evaluated.

The objective of this analysis was therefore to determine the interindividual variability in the bioavailability of gabapentin enacarbil using PK data from 6 phase I studies and to compare these results with those for other formulations of gabapentin reported in the literature.

## MATERIALS AND METHODS

PK parameters and bioavailability data for gabapentin after a single oral dose of gabapentin enacarbil 1200 mg were analyzed using data from 6 phase I PK studies (XP-022, XP-044, XP-057, XP-067, XP-068, and XP-087) enrolling healthy adults. These studies have been described previously.^15,19–21^ Each study protocol and informed consent form were approved by the institutional review board at each site. Each study was conducted in accordance with the tenets of the International Conference on Harmonization Good Clinical Practice Guidelines and the guiding principles of the Declaration of Helsinki.

### Subjects

All subjects enrolled in the 6 phase I studies were healthy adults, as determined by the absence of clinically significant medical conditions, physical examination, clinical laboratory results, and electrocardiograms at screening. The included subjects were aged 18–80 years (XP-022, XP-044, and XP-057) or 18–55 years (XP-067, XP-068, and XP-087).

The key exclusion criteria included a history or presence of GI disease or any abnormality or illness that could affect the absorption, distribution, metabolism, or elimination of the study drug. Subjects with creatinine clearance <60 mL/min (XP-022, XP-044, and XP-057) or <80 mL/min (XP-067, XP-068, and XP-087), as determined by the Cockcroft–Gault formula, were excluded from the study. Across all studies, female subjects were required to have a negative pregnancy test at screening. Those with childbearing potential agreed to use a clinically accepted form of birth control throughout the study. All subjects provided written informed consent for participation.

### Study Designs

#### XP-022

XP-022 was a randomized crossover study to assess oral XP13512 sustained-release (SR) tablets (gabapentin enacarbil) or commercial gabapentin in healthy adults.^19^ A total of 12 subjects were randomized to one of 3 treatment sequences; ABC, BAC, or CAB, where (A) consisted of a single dose of XP13512 SR (gabapentin enacarbil) 1200 mg (2 600-mg SR tablets; MDS Pharma Services, Tampa, FL) in a fasted state, (B) consisted of a single dose of XP13512 SR (gabapentin enacarbil) 1200 mg (2 600-mg SR tablets) in a fed state, and (C) consisted of a single dose of 600 mg commercial gabapentin (2 300-mg capsules) in a fasted state. Gabapentin enacarbil 1200 mg is equivalent to approximately 625 mg of gabapentin.^19^ Subjects received all 3 doses with a 7-day washout between each dose. The PK data from 10 subjects after a single 1200-mg dose of gabapentin enacarbil in a fed state were used for this analysis.

#### XP-044

XP-044 was a randomized, crossover study aiming to assess 3 different doses of oral XP13512 SR tablets (gabapentin enacarbil) in healthy adults.^19^ A total of 36 subjects were randomized to receive one of 3 single doses of XP13512 SR (Cardinal Health, Somerset, NJ; 1 300-mg SR tablet, 1 600-mg SR tablet, or 2 600-mg SR tablets) in fasted and fed states, with a 7-day washout between each dose. For this analysis, the PK data from 12 subjects after a single 1200-mg dose in a fed state were used.

#### XP-057

XP-057 was a randomized crossover study to assess 2 oral SR formulations of XP13512 (gabapentin enacarbil) in healthy adults.^15^ A total of 12 subjects were randomized to 1 of 2 formulation sequences: A then B or B then A. Formulation A was a single dose of gabapentin enacarbil 1200 mg (2 600-mg MDS SR tablets; MDS Pharma Services), and formulation B was a single dose of gabapentin enacarbil 1200 mg (2 600-mg Patheon SR tablets; Patheon, Cincinnati, OH), with a 7-day washout between treatment periods. All doses of gabapentin enacarbil were received by the subjects in a fed state. PK data obtained from 12 subjects after a single dose of formulation B were used for this analysis. Data from formulation B were used for this study to assess the marketed formulation (Horizant) wherever possible.

#### XP-067

XP-067 was an open-label, 3-period, drug-interaction study to assess the PK of XP13512 SR tablets (gabapentin enacarbil) coadministered with naproxen in healthy adults.^20^ Subjects received XP13512 SR (gabapentin enacarbil) 1200 mg (2 600-mg SR tablets; Patheon) once daily for 5 days in period 1 (days 1–5), naproxen (Naprosyn; Roche, Basel, Switzerland) 500 mg twice daily (2 250-mg tablets) for 5 days in period 2 (days 6–10), and both XP13512 SR (gabapentin enacarbil) 1200 mg once daily (2 600-mg SR tablets) and naproxen 500 mg twice daily (2 250-mg tablets) for 5 days in period 3 (days 11–15). All doses of gabapentin enacarbil were received by the subjects in a fed state. PK sampling was conducted on the last day of each treatment period. PK data for gabapentin enacarbil alone were available from 11 subjects and were used for this analysis.

#### XP-068

XP-068 was an open-label, drug-interaction study of XP13512 SR tablets (gabapentin enacarbil) coadministered with cimetidine (Tagamet; GlaxoSmithKline, Brentford, United Kingdom) in healthy adults.^20^ In this 3-period crossover study, 12 subjects received gabapentin enacarbil 1200 mg (2 600-mg SR tablets; Patheon) once daily for 4 days in period 1 (days 1–4), cimetidine 400 mg 4 times daily for 4 days in period 2 (days 5–8), and both gabapentin enacarbil 1200 mg once daily and cimetidine 400 mg 4 times daily for 4 days in period 3 (days 9–12). All doses of gabapentin enacarbil were received by the subjects in a fed state. PK sampling was conducted on the last day of each treatment period. PK data for gabapentin enacarbil alone were available from 12 subjects and were used for this analysis.

#### XP-087

XP-087 was a randomized, open-label, food-effect comparison study of XP13512 SR tablets (gabapentin enacarbil) in healthy adults.^21^ In this 4-period crossover study, 12 subjects each received a single 1200-mg dose of gabapentin enacarbil (2 600-mg SR tablets; Patheon) after each of the following 4 different meal types: an overnight fast, a low-fat breakfast, a moderate-fat breakfast, or a high-fat breakfast meal, with a washout period of 5–7 days between periods. PK data from 9 subjects following the moderate-fat breakfast meal were used in the current analysis.

### Sample Collection

Blood samples were collected predose (up to 1 hour before study drug administration) and at the following hours postdose: 0.5, 1, 1.5 (XP-022, XP-068, XP-087 only); 2, 3, 4, and 5 (all except XP-022); 6, 7 (XP-087 only); 8, 10 (all except XP-022); 12, 13 (XP-068 only); 14 (XP-068 and XP-087 only); 15 (XP-068 only); 16 (XP-067 and XP-068 only); 18, 24, 30 (XP-087 only); 36 (all except XP-067 and XP-068); and 168 hours (XP-022 and XP-044 only). For whole-blood evaluations, 5–6-mL blood samples were collected in tubes containing dipotassium salt of ethylenediaminetetraacetic acid, and 2 1 mL samples were immediately quenched with 3 mL methanol and stored in polypropylene tubes at −70°C until analysis. For plasma evaluation, 6–10 mL blood samples were collected in tubes containing dipotassium salt of ethylenediaminetetraacetic acid and centrifuged for 15 minutes at 2000 g at 4°C. Two aliquots of plasma were transferred to polypropylene tubes, and samples were stored at or below −20°C until being transported for analysis.

Urine samples were collected before dosing (for all studies except XP-067 and XP-068) and with the following intervals: 0–4 hours (0–3 hours for XP-068), 4–8 hours (3–6 hours for XP-068), 8–12 hours (6–12 hours for XP-068), 12–24 hours, and 24–36 hours (all except XP-067 and XP-068). The total urine output was measured at each interval, and 2 aliquots of each sample were stored at or below −20°C until being transported for analysis.

### PK Analysis

PK measurements were performed using samples of whole blood (XP-067) or plasma (XP-022, XP-044, XP-057, XP-068, and XP-087) and urine. Bioavailability was determined using the urine samples. All other PK analyses were performed using plasma values where available; blood values were used when plasma values were not available and converted to plasma by multiplying each subject's PK value by 1.26. Samples were analyzed for gabapentin concentrations at MDS Pharma Services (Lincoln, NEXP-022) or XenoPort (Santa Clara, CA; XP-044, XP-057, XP-067, XP-068, XP-087) using validated high-pressure liquid chromatography coupled with tandem mass spectrometry. As previously described, quenched blood or plasma samples were injected on a Zorbax XDB (3.5 μm; 50 × 4.6 mm; Agilent Technologies, Santa Clara, CA) or Phenomenex Hydro-RP column (4 μm; 50 × 4.6 mm; Phenomenex, Torrance, CA) operated at 30°C. The mobile phases were 0.1% formic acid in water (A) and acetonitrile (B). The gradient was set at time 0 minutes, 95% A; 0.5 minutes, 95% A; 1.8 minutes, 5% A; 3.0 minutes, 5% A; 3.5 minutes, 2% A; and 4.1 minutes, 95% A; until 6 minutes. The flow rate was 1000 μL/min for the blood and 1200 μL/min for the plasma. Detection was performed using a Sciex API 2000 mass spectrometric detector (Applied Biosystems, Foster City, CA) using a positive-ion, multiple-reaction monitoring mode with ion transitions (m/z) of 172.1/137.2, gabapentin and 200/154 for L-4-chlorophenylalanine (internal standard for gabapentin).

The bioanalytical method for determination of gabapentin in blood was validated over a concentration range of 50 to 12 500 ng/mL using quality control samples at 150, 1000, and 9500 ng/mL. The method for determination of gabapentin in plasma was validated over the concentration range of 80 to 10,000 ng/mL using quality control samples at 240, 1600, and 7500 ng/mL. Intrabatch precision (percent coefficient of variation [CV]) and accuracy (deviation from theoretical values) were ≤13% and ≤104%, respectively, for blood, and ≤2.8% and ≤104%, respectively, for plasma. The interbatch precision and accuracy were ≤10% and ≤102%, respectively, for blood, and ≤5.8% and ≤104%, respectively, for plasma.^15^

Urine samples were analyzed using validated liquid chromatography–tandem mass spectrometry, similar to that for blood/plasma. The method for gabapentin in urine was linear over the concentration range of 50 to 12 500 ng/mL. The intrabatch precision was <5%, and the accuracy ranged from 96% to 102%. The interbatch precision was <5%, and accuracy was 98%–105%.

### Statistical Analysis

The concentration data for gabapentin were analyzed by noncompartmental methods using WinNonlin version 4.1 (Pharsight, Mountain View, CA). The maximum concentration (C_max_) and time to C_max_ were obtained by direct interpretation of the graphical concentration data. The terminal elimination half-life was obtained by linear regression of 3 or more log-transformed data points in the terminal phase. Systemic drug exposure was assessed using the area under the plasma concentration–time curve (AUC) and quantified using the linear trapezoidal method over the dosing interval. For single-dose studies, AUC values extrapolated from time 0 to infinity (AUC_0–∞_) were calculated using the equation ${\text{AUC}}_{0-\infty }={\text{AUC}}_{\left(0-{\text{T}}_{\text{last}}\right)}+{\text{C}}_{\text{last}}/\lambda_{\text{z}}$, where T_last_ is the time of the last quantifiable concentration (C_last_) and λ_z_ is the rate constant of the terminal elimination phase. The AUC value in the steady state was calculated for multiple-dose studies.

Bioavailability was expressed as the percentage of the oral dose excreted as gabapentin (based on a theoretical dose of 625 mg of gabapentin per 1200 mg of gabapentin enacarbil dose) using the percentage dose excreted (%F) = 100 * (Ae/D), where Ae is the total amount of gabapentin excreted during each urine collection period and D is the administered dose of gabapentin enacarbil expressed in mg equivalents of gabapentin. After absorption, gabapentin is excreted in urine without further metabolism; therefore, the percentage of the administered dose of gabapentin equivalents recovered in urine is an accurate reflection of the percentage of dose absorbed and converted to gabapentin (ie, bioavailability as gabapentin). The use of urinary recovery data requires accurate measurement of urine volume for each collection period. Subjects with inaccurate urine collection measurements were excluded from the PK analyses. The total amount of gabapentin excreted during each urine collection period (Ae) was calculated using the equation Ae_(t1–t2)_ = C_(t1–t2)_ × V_(t1–t2)_, where Ae_(t1–t2)_ is the amount excreted in mg over the time interval t1–t2, C_(t1–t2)_ is the concentration in mg/mL of gabapentin in the urine collected over this interval, and V_(t1–t2)_ is the total volume of the urine sample in milliliters. The total amount excreted (Ae) over 36 hours (Ae_[0–36]_) for single-dose studies, or over 1 dosing interval at steady state (Ae_[0–tau]_) for multiple-dose studies, was calculated as the sum of the amounts excreted in all intervals. The mean, SD, and CV were calculated by study as well as across all 6 studies combined. An analysis of the data pooled from individual subjects across studies was further conducted using R software version 4.04 (Foundation for Statistical Computing, Vienna, Austria).

## RESULTS

### Subjects

The baseline characteristics of the participants were generally comparable across the 6 studies (Table 1). Most subjects were male, whereas subjects in XP-022, XP-044, and XP-057 were on average older (mean age range: 41–53 years) than those in XP-067, XP-068, and XP-087 (mean age range: 25–31 years).

**TABLE 1.** Baseline Characteristics of Participants by Study

Characteristic	XP-022 (n = 12)	XP-044 (n = 12)	XP-057 (n = 12)	XP-067 (n = 12)	XP-068 (n = 12)	XP-087 (n = 12)
Age, years	53 (19)	46 (17)	41 (16)	31 (12)	25 (6)	30 (12)
Male, n (%)	7 (58)	6 (50)	8 (67)	8 (67)	11 (92)	8 (67)
Height, cm	173 (10)	170 (7)	NR	171 (7)	175 (8)	155–192*
Weight, kg	75 (9)	77 (13)	NR	72 (12)	75 (9)	61–94*
BMI, kg/m^2^	25 (2)	26 (3)	NR	24 (NA)	NR	20–29*

All values are mean (SD) unless otherwise noted.

* Minimum–maximum.

BMI, body mass index; NA, not available; NR, not reported.

### PK Outcomes

**FIGURE 2.**
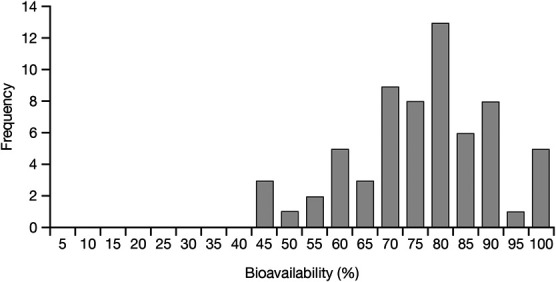
Frequency distribution of bioavailability values across studies after one oral dose of gabapentin enacarbil 1200 mg in healthy subjects. The *x*-axis values represent the upper bounds of 5% intervals (eg, 45% refers to bioavailability between 40% and 45%).

In general, the PK parameters were similar across all 6 studies (Table 2). Mean C_max_ of gabapentin ranged from 6.4 to 7.9 μg/mL and were achieved from 5.2 to 8.2 hours after dosing. The mean AUC_0–∞_ (or AUC value at steady state for XP-067 and XP-068) ranged from 70.8 to 109.4 μg·h/mL. The mean gabapentin bioavailability from oral gabapentin enacarbil ranged from 64.8% to 82.9% across all 6 studies (Table 2). When the bioavailability values of each subject from all 6 studies were pooled, the mean was 74.1% (SD = 14.1; CV = 19.1%). The distribution of individual gabapentin bioavailabilities ranged from 42% to 100% (Fig. 2).

**TABLE 2.** Gabapentin PK Parameters Across Studies After an Oral Dose of 1200 mg Gabapentin Enacarbil in Healthy Individuals

Parameter	XP-022 (n = 10)	XP-044 (n = 12)*	XP-057† (n = 12)	XP-067‡ (n = 11)	XP-068‡ (n = 12)	XP-087§ (n = 9)	Overall (N = 66)¶
C_max_, μg/mL							
Mean (SD)	7.9 (1.6)	7.6 (1.7)	7.6 (1.3)	7.8 (1.6)	7.0 (1.2)	6.4 (1.2)	7.4 (1.5)
CV, %	19.7	21.8	16.6	20.3	17.0	18.0	19.5
Geo. mean	7.8	7.4	7.5	7.6	6.9	6.3	7.3
T_max_, h							
Mean (SD)	8.2 (2.2)	7.9 (2.2)	7.4 (2.2)	5.2 (1.1)	5.6 (0.5)	7.3 (2.3)	6.9 (2.1)
CV, %	26.8	27.7	29.0	20.8	9.5	31.8	30.7
Geo. mean	8.0	7.7	7.2	5.1	5.6	7.1	6.6
AUC_0–∞,_‖ μg·h/mL							
Mean (SD)	109.4 (27.6)	92.4 (13.0)	98.8 (19.0)	79.8 (14.1)	70.8 (10.0)	75.9 (6.6)	87.8 (20.7)
CV, %	25.2	14.0	19.2	17.7	14.2	8.7	23.6
Geo. mean	106.3	91.6	97.2	78.7	70.1	75.6	85.7
Bioavailability, %							
Mean (SD)	74.5 (7.3)	82.1 (12.2)	82.9 (13.5)	71.9 (12.5)	67.1 (13.2)	64.8 (16.9)	74.1 (14.1)
CV, %	9.8	14.9	16.2	17.4	19.7	26.0	19.1
Geo. mean	74.2	81.2	81.9	70.8	65.8	62.8	71.6

Plasma values were used when available; blood values were otherwise used and converted to plasma by multiplying each subject's PK value by 1.26.

* n = 10 for bioavailability.

† Data from the Patheon formulation were used for the analysis.

‡ PK sampling occurred on the final day of a 5-day (XP-067) or 4-day (XP-068) administration of gabapentin enacarbil 1200 mg once daily.

§ Data from moderate-fat breakfast meals were used for this analysis.

¶ N = 64 for bioavailability.

‖ Area under the plasma concentration time curve at steady state for XP-067 and XP-068.

AUC0–∞, area under the plasma–concentration time curve extrapolated from time zero to infinity; Cmax, maximum concentration; CV, coefficient of variation; PK, pharmacokinetic; T_max_, time to maximum concentration.

## DISCUSSION

In this analysis of 6 gabapentin enacarbil phase I studies, the PK parameters of gabapentin absorption after gabapentin enacarbil administration were consistent across all studies. The mean bioavailability of gabapentin from the prodrug was high and displayed less interindividual variability than reported for oral gabapentin administration.^10,17,18^

**FIGURE 3.**
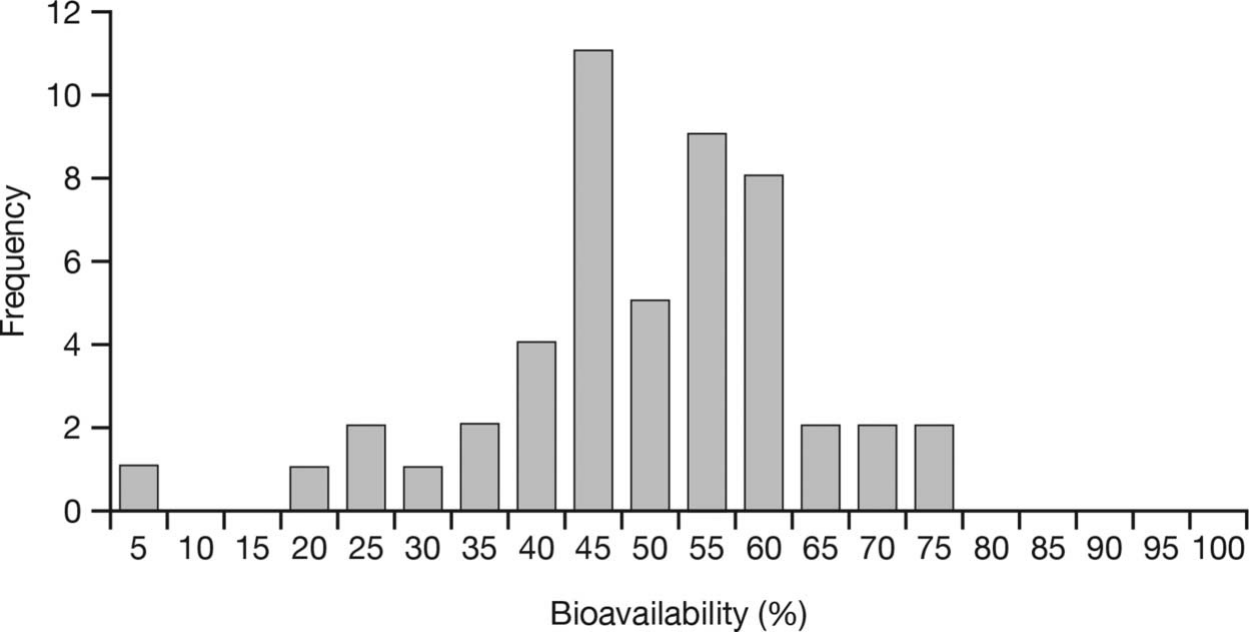
Frequency distribution of bioavailability values after a single-dose administration of gabapentin 600 mg to 50 healthy volunteers. Reprinted from Gidal BE, Radulovic LL, Kruger S, et al. Inter- and intra-subject variability in gabapentin absorption and absolute bioavailability. *Epilepsy Res*. 2000;40:123–127. Copyright 2000, with permission from Elsevier.

Variations in the bioavailability of gabapentin from gabapentin enacarbil have not been evaluated previously. Although gabapentin PK after administration of gabapentin immediate release, gabapentin gastroretentive, and gabapentin enacarbil was compared in a 3-way head-to-head study, no urine measurements were conducted and the bioavailability of gabapentin could not be included in the comparison.^12^ In this current analysis, gabapentin bioavailability after an oral 1200-mg dose of gabapentin enacarbil in 64 subjects ranged from 42% to 100%, with a mean of 74.1% and an intersubject CV of 19.1%. By contrast, a previous study showed greater interindividual variation in gabapentin bioavailability after a single oral 600-mg dose of oral gabapentin among 50 healthy subjects, ranging from 5% to 74% with a mean of 49.3% and an intersubject CV of 27.6% (Fig. 3).^10^ This difference is consistent with the respective absorption pathways of gabapentin enacarbil and gabapentin; gabapentin bioavailability is likely to be more predictable after administration of gabapentin enacarbil because of the absorption of the prodrug by multiple high-capacity transporters located throughout the GI tract and increased permeability compared with gabapentin.^13,14^ Indeed, the linear dose–exposure relationship for gabapentin enacarbil and the nonlinear nature of that for gabapentin have been previously demonstrated in a meta-analysis that used a model-based approach to address issues associated with between-study variability.^17^

The PK profile of gabapentin after oral dosing is variable and unpredictable. In a comparison of the PK profiles of plasma gabapentin after the administration of gabapentin immediate release, gabapentin gastroretentive, and gabapentin enacarbil, PK parameters varied widely between formulations despite being measured in the same set of subjects.^12^ Furthermore, distinct differences in PK parameters were observed between formulations of oral immediate-release gabapentin previously accepted to be bioequivalent.^22^

A limitation of this study was that the study population comprised only healthy subjects. Thus, our results should be interpreted with caution in the elderly or in patients with altered renal or GI function, and further investigation is warranted in patients with RLS or PHN. The impact of interpatient variability in renal function and comorbidities on the overall bioavailability, efficacy, and safety of gabapentin in patients with RLS or PHN is of clinical interest.

## CONCLUSIONS

The prodrug gabapentin enacarbil demonstrated low interindividual variability for the bioavailability of the active drug in healthy subjects and may thus offer a more consistent and predictable absorption across clinically relevant dose levels than other gabapentin formulations. Further studies are needed to determine whether this low variability in bioavailability translates into clinical practice.
